# Efficacy and safety of anlotinib as maintenance treatment in extensive-stage small cell lung cancer: a single-armed single center retrospective study

**DOI:** 10.3389/fonc.2024.1462581

**Published:** 2025-01-14

**Authors:** Jin Xiong, Lei Xia

**Affiliations:** ^1^ Department of Cancer Center, The Second Affiliated Hospital of Chongqing Medical University, Chongqing, China; ^2^ Tianjin Key Laboratory of Radiation Medicine and Molecular Nuclear Medicine, Institute of Radiation Medicine, Chinese Academy of Medical Sciences and Peking Union Medical College, Tianjin, China; ^3^ Department of Radiation Oncology, Tianjin Medical University Cancer Institute and Hospital, National Clinical Research Center for Cancer, Tianjin’s Clinical Research Center for Cancer, Key Laboratory of Cancer Prevention and Therapy, Tianjin, China

**Keywords:** extensive-stage small cell lung cancer, effectiveness, anlotinib, safety, adverse (side) effects

## Abstract

**Introduction:**

Patients with extensive-stage small cell lung cancer (ES-SCLC) have a poor Q6 prognosis and there is no standard protocol for maintenance treatment. Anlotinib as a third-line or beyond therapy for ES-SCLC was proved to be effective.

**Methods:**

We retrospectively screened of patients with ES-SCLC who started receiving anlotinib as first-line or second-line therapy at the Second Affiliated Hospital of Chongqing Medical University from November 2018 to December 2022. 30 patients treated with anlotinib based combination therapy and subsequent maintenance therapy were included. The primary study endpoint was progression-free survival (PFS) and the secondary study endpoints were overall survival (OS), clinical response and adverse events (AEs).

**Results and discussion:**

In 30 ES-SCLC patients, the median PFS and OS were 7.2 months and 17.6 months respectively. The ORR and DCR were 50.0% (15/30) and 86.7% (26/30) respectively. The median PFS was 8.2 months and 5.6 months for patients who received synchronized immunotherapy or chemotherapy. The median OS was 20.1 months and 15.1 months for patients who received synchronized immunotherapy or chemotherapy. The median time to intracranial progression (TTP) was 7.2 months for patients who were without brain metastases before receiving anlotinib. No unexpected AEs were reported. Grade 3-4 adverse events were reported in 10 patients (33.3%). No treatment-related deaths occurred during this study. Our study has indicated the good efficacy and safety about the application of anlotinib in the maintenance therapy in the first-line or second-line treatment of ES-SCLC and it can also achieve good intracranial control.

## Introduction

Small cell lung cancer (SCLC) is a high-grade neuroendocrine carcinoma that accounts for approximately 15% of all lung cancers, which occurs primarily in current or former smokers (1). With an exceptionally poor prognosis, SCLC is regarded as the most malignant type of all lung cancers and two-thirds of SCLC patients present extensive stage combined with distant metastases at the time of initial diagnosis (2). Dual chemotherapy of platinum (cisplatin or carboplatin) and etoposide is the standard first-line regimen for patients with extensive-stage small cell lung cancer (ES-SCLC) and they show a high response rate after the treatment (3). However, the median progression-free survival (PFS) is only 2-3 months and the median overall survival (OS) is about 10 months as patients often show drug resistance and have a high relapse rate (4). Meanwhile, patients with SCLC are insensitive to second-line and subsequent treatments, the 5-year survival rate is less than 5%. Therefore, numerous studies were conducted to improve the therapeutical outcomes for patients with ES-SCLC in recent years.

Recently, IMpower133 trial evaluated the efficacy of PD-L1 inhibitors of atezolizumab in platinum-etoposide chemotherapy for ES-SCLC patients, which showed improvements in OS (12.3m vs. 10.3m) and PFS (5.2m vs. 4.3m) comparing with chemotherapy alone (5). Meanwhile, another similar study focused on the durvalumab plus chemotherapy comparing chemotherapy alone also proved prolonged OS (12.3m vs. 10.3m) (2). However, the addition of PD-L1 inhibitors to chemotherapy for SCLC was not as effective as those for non-small cell lung cancer (NSCLC) (6). Meanwhile, once standard treatment stops, many patients will experience rapid recurrence or metastases which lead to rapid disease deterioration and death. Therefore, it is urgent to find more effective and safe maintenance treatment methods.

The formation of neovascularization plays an important role in the growth, invasion and metastases of malignant tumors. SCLC highly expresses vascular endothelial growth factor (VEGF) and its receptors, stem cell factors and c-Kit (c-Kit) (7). Therefore, anti-angiogenesis becomes a potential therapeutic strategy. There have been several studies exploring the application of pazopanib, sunitinib and sorafenib in patients with ES-SCLC, but their efficacy is not satisfactory.

Anlotinib hydrochloride is a type of tyrosine kinase inhibitors (TKIs) targeting vascular endothelial growth factor receptor (VEGFR), platelet-derived growth factor receptor (PDGFR), fibroblast growth factor receptor (FGFR) and stem cell factor receptor (c-Kit), which can inhibit both tumor angiogenesis and tumor growth simultaneously (8). Several clinical trials have demonstrated the clinical benefits of anlotinib in various tumors such as locally advanced or metastatic medullary thyroid cancer, metastatic renal cell carcinoma (9, 10). ALTER1202 study observed that anlotinib hydrochloride group gained survival benefit comparing with the placebo group in PFS (4.1m vs. 0.7m) and OS (7.3m vs. 4.9m) in the third-line and further-line therapy of patients with SCLC (11). Two recent phase II studies focused on the first-line treatment of ES-SCLC also revealed the good efficacy and safety of anlotinib combined with platinum-based chemotherapy (12, 13).

Therefore, we conducted this retrospective clinical study to evaluate the effectiveness and safety of anlotinib as maintenance therapy in combination with immunotherapy or chemotherapy in patients with ES-SCLC.

## Methods

### Patients

Research subjects are patients with extensive-stage small cell lung cancer who started receiving anlotinib as first-line or second-line maintenance therapy at the Second Affiliated Hospital of Chongqing Medical University from November 1, 2018 to December 1, 2022. Retrospectively screening of all patients with extensive-stage small cell lung cancer hospitalized at the Second Affiliated Hospital of Chongqing Medical University. Inclusion criteria were: (1) Pathologically confirmed inoperable SCLC; (2) Treated with anlotinib in combination with other regimens during first-line or second-line therapy. (3) At least one lesion that can be measured according to the solid tumor efficacy evaluation criteria (RECIST 1.1). (4) Patients are between 18 and 85 years of age. (5) ECOG PS score of 0-2. (6) Survival ≥ 3 months.

Exclusion criteria: (1) combination of other types of malignancies; (2) uncontrollable hypertension and arrhythmias, risk of hemoptysis and severe hepatic and renal insufficiency; (3) uncontrolled severe infections; (4) poor adherence to treatment and easy loss of follow-up. This study was approved by the Ethics Committee of the Second Affiliated Hospital of Chongqing Medical University in accordance with the principles of the Declaration of Helsinki. Because of the retrospective nature of this study, informed consent was not required.

### Therapeutic schemes

Patients were given a 21-day cycle of anlotinib (provided by Zhengda Tianqing) at a dose of 12 mg/day from day 1 to day 14, immunotherapy or chemotherapy was given simultaneously for 4-6 cycles. Anlotinib maintenance treatment was then continued until disease progression or unacceptable adverse effects (AEs) occurred. Reduction or temporary discontinuation of the drug was allowed depending on patient tolerability. The initial dose of 12 mg/d of anlotinib may be reduced to 10 mg and then to 8 mg, and may be readministered after AEs are tolerated.

### Data collection and outcome assessment

The medical records of each patient were retrospectively collected which include patient demographic and clinical background, blood biochemical data, treatment modality, treatment efficacy and treatment-related AEs. The primary study endpoint was PFS and the secondary study endpoints were OS, objective remission rate (ORR), disease control rate (DCR), intracranial progression-free time (TTP) and drug safety. The investigators assessed tumor response according to RECIST 1.1. Efficacy was evaluated as complete remission (CR), partial remission (PR), stable disease (SD) or progressive disease (PD). ORR and DCR were defined as the proportion of patients achieving CR or PR and CR, PR or SD respectively. All adverse events severity were graded according to the National Cancer Institute’s Common Terminology Criteria for Adverse Events (CTCAE) version 5.0.

### Follow-up

Follow-up began after treatment with anlotinib with imaging evaluations every 6-8 weeks and regular monthly follow-up of patient condition was conducted through the outpatient and inpatient system or by phone. The last follow-up date was March 1, 2023. The median follow-up time was 18.4 months.

### Statistical analysis

Categorical variables were expressed as frequencies (proportions) and continuous variables were expressed as means ± standard deviations (SD). Median and estimated 95% confidence intervals (CI) for PFS and OS were calculated using the Kaplan-Meier method. The log-rank test was used to compare survival differences among subgroups of patients. Cox proportional risk models were used to estimate the risk factors associated with patient survival. The significance level of statistical tests was set at *p* < 0.05. Survival curves were generated using Prism 8.0 (GraphPad software, La Jolla, CA, USA). Statistical analysis was performed using SPSS 27.0.

## Results

**Figure 1:**
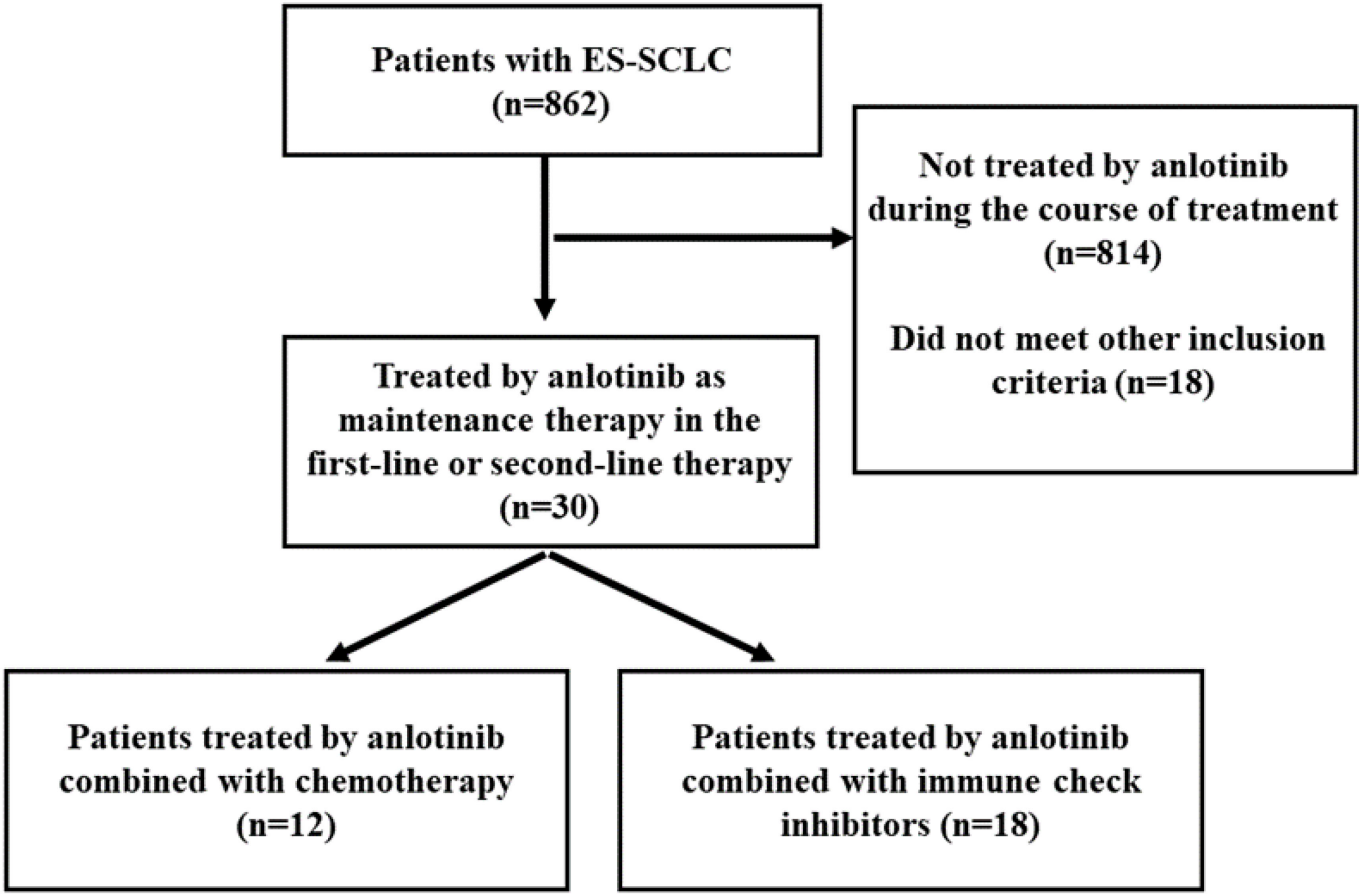
Study subject profile.

A total of 862 patients were screened from November 1, 2018 to December 1, 2021, and finally 30 patients were found eligible (**Figure 1**).

### Patient characteristics

This study included 30 participants whose demographic and clinical backgrounds were detailed in **Table 1**. 21 of the 30 patients (70.0%) were male. The median age was 64 with a range of 51-82. 14 patients (46.7%) were younger than 65 of age (14/30). The admission ECOG PS score was less than 3 in all patients, 0 in 13.3% (4/30), 1 in 40% (12/30), and 2 in 46.7% (14/30). Patients with a history of previous smoking accounted for 70% (21/30). Patients with a history of previous hypertension accounted for 26.7% (8/30). Patients with initial treatment as neoplasm staging III accounted for 30% (9/30) and 70% (21/30) of patients with neoplasm staging IV. The regional lymph nodes were the most common site of metastases, accounting for 90% (27/30). 13.3% (4/30) and 86.7% (26/30) of the patients received anlotinib in the first-line and second-line therapy respectively. Patients treated with immune checkpoint inhibitors in combination with anlotinib accounted for 60% (18/30), patients treated with chemotherapy in combination with anlotinib accounted for 40% (12/30).

**Table 1:** Demographics and baseline characteristics of patients included.

Characteristics	No. (%) or value
Sex	
Female, n (%)	9 (30.0)
Male, n (%)	21 (70.0)
Age, years	64.47 ± 8.66
Age group, years	
<65 years	14 (46.7)
≥65 years	16 (53.3)
ECOG PS at baseline, n (%)	
0	4 (13.3)
1	12 (40.0)
2	14 (46.7)
Smoking history, n (%)	
No	9 (30.0)
Yes	21 (70.0)
Hypertension history, n (%)	
No	22 (73.3)
Yes	8 (26.7)
Neoplasm staging, n (%)	
III	9 (30.0)
IV	21 (70.0)
Metastatic sites at baseline	
Lymph node	27 (90.0)
Pleura	12 (40.0)
Bone	5 (16.7)
Bilateral lung	2 (6.7)
Liver	4 (13.3)
Brain	1 (3.4)
Anlotinib in therapy line, n (%)	
first line	4 (13.3)
second line	26 (86.7)
Anlotinib combined with Immunotherapy, n (%)	
No	12 (40.0)
Yes	18 (60.0)

### Treatment pattern

The anlotinib treatment pattern is shown in **Table 2**. Systemic treatment models included anlotinib in combination with chemotherapy (40.0%, n=12) and anlotinib in combination with immunotherapy (60.0%, n=18). Chemotherapeutic agents were mainly albumin-bound paclitaxel, etoposide combined with cisplatin, irinotecan and temozolomide. Immunotherapeutic agents included sintilimab, atezolizumab and camrelizumab. For local treatment, 93.3% (n=28) of patients were combined with lung radiotherapy, 40.0% (n=12) with brain radiotherapy and 23.3% (n=7) with bone radiotherapy.

**Table 2:** Anlotinib treatment pattern.

Category or variable	No. (%) or value
No. of patients	30 (100.0)
Treatment line	
1st line	4 (13.3)
2nd line	26 (86.7)
Systemic treatment	
Anlotinib plus chemotherapy	12 (40.0)
Albumin-bound paclitaxel	2 (6.7)
Etoposide plus cisplatin	4 (13.3)
Irinotecan	5 (16.7)
Temozolomide	1 (3.3)
Anlotinib plus immunotherapy	18 (60.0)
Sintilimab alone	10 (33.3)
Atezolizumab alone	6 (20.0)
Camrelizumab alone	2 (6.7)
Radiotherapy history	
Lung radiotherapy history	28 (93.3)
Brain radiotherapy history	12 (40.0)
Bone radiotherapy history	7 (23.3)
Others	4 (13.3)

### Best overall response

**Figure 2:**
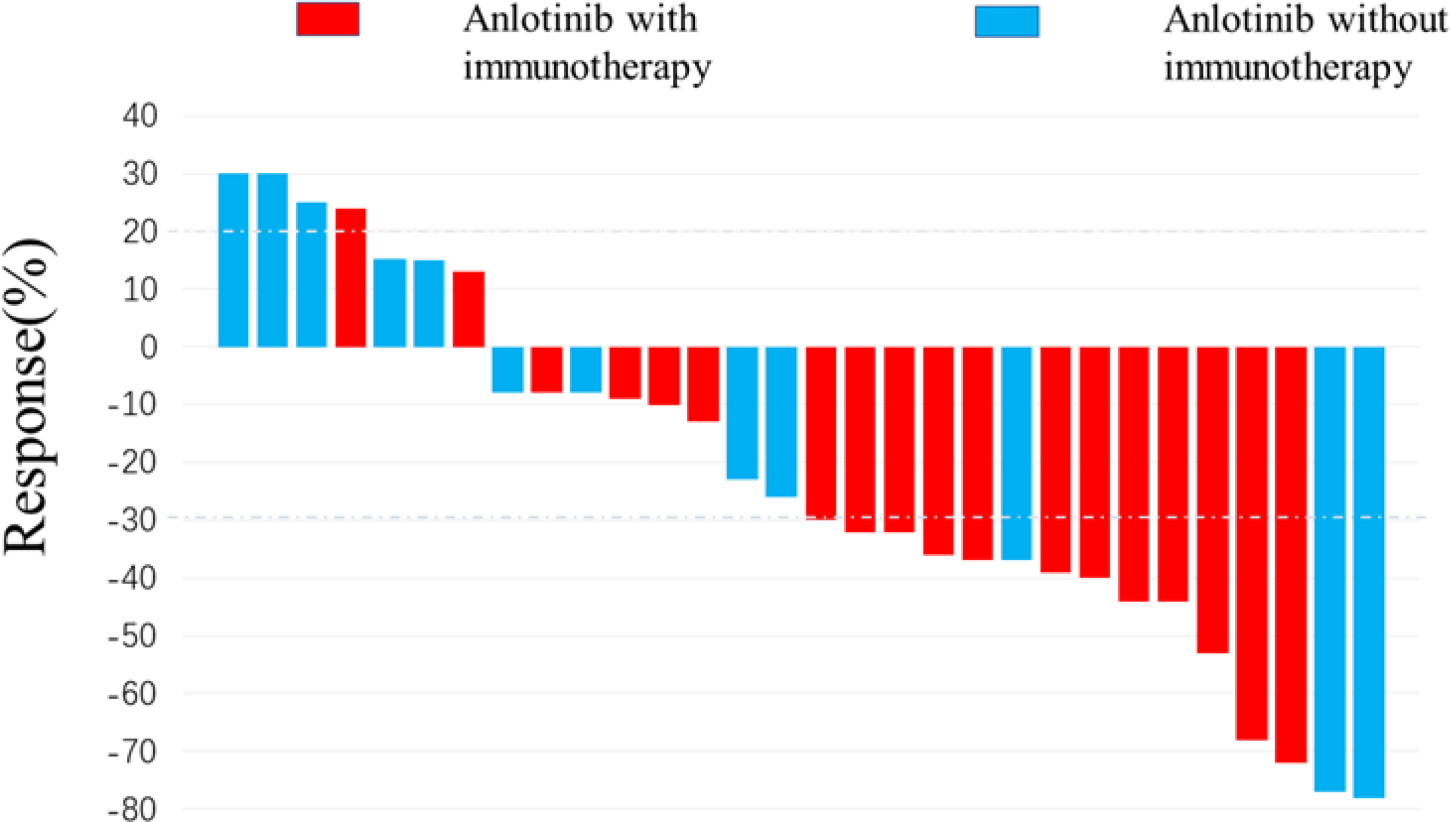
Waterfall plot for best response. 15 patients achieved target tumor shrinkage greater than 30%. The color indicates the types of therapy patterns.

**Figure 2** depicts the optimal change from baseline in detectable target lesions in 30 patients. Among the 30 patients in this study, the incidence of CR, PR, SD and PD were 0% (0/30), 50.0% (15/30), 36.67% (11/30) and 13.4% (4/30). The ORR and DCR were 50.0% (15/30) and 86.7% (26/30) respectively. The differences in SD, PD and DCR between the immunotherapy and non-immunotherapy groups were not statistically significant, but the PR rates and ORR were higher in the combined immunotherapy group than in the non-combined immunotherapy group (66.7% vs. 25.0%, *p*=0.034) (**Table 3**).

**Table 3:** Tumor response.

Category	With immunotherapy(n=18)	Without immunotherapy(n=12)	*p*	All patients(n=30)
CR (%)	0 (0)	0 (0)		0 (0)
PR (%)	12 (66.7)	3 (25.0)	0.034	15 (50.0)
SD (%)	5 (33.3)	6 (50.0)	0.266	11 (36.7)
PD (%)	1 (5.6)	3 (25.0)	0.274	4 (13.3)
ORR	66.7% (12/18)	25.0% (3/12)	0.034	50.0% (15/30)
DCR	94.4% (17/18)	75.0% (9/12)	0.274	86.7% (26/30)

CR, complete response; DCR, disease control rate; ORR, objective response rate; PD, progressive disease; PR, partial response; SD stable disease.

### Treatment outcomes

**Figure 3:**
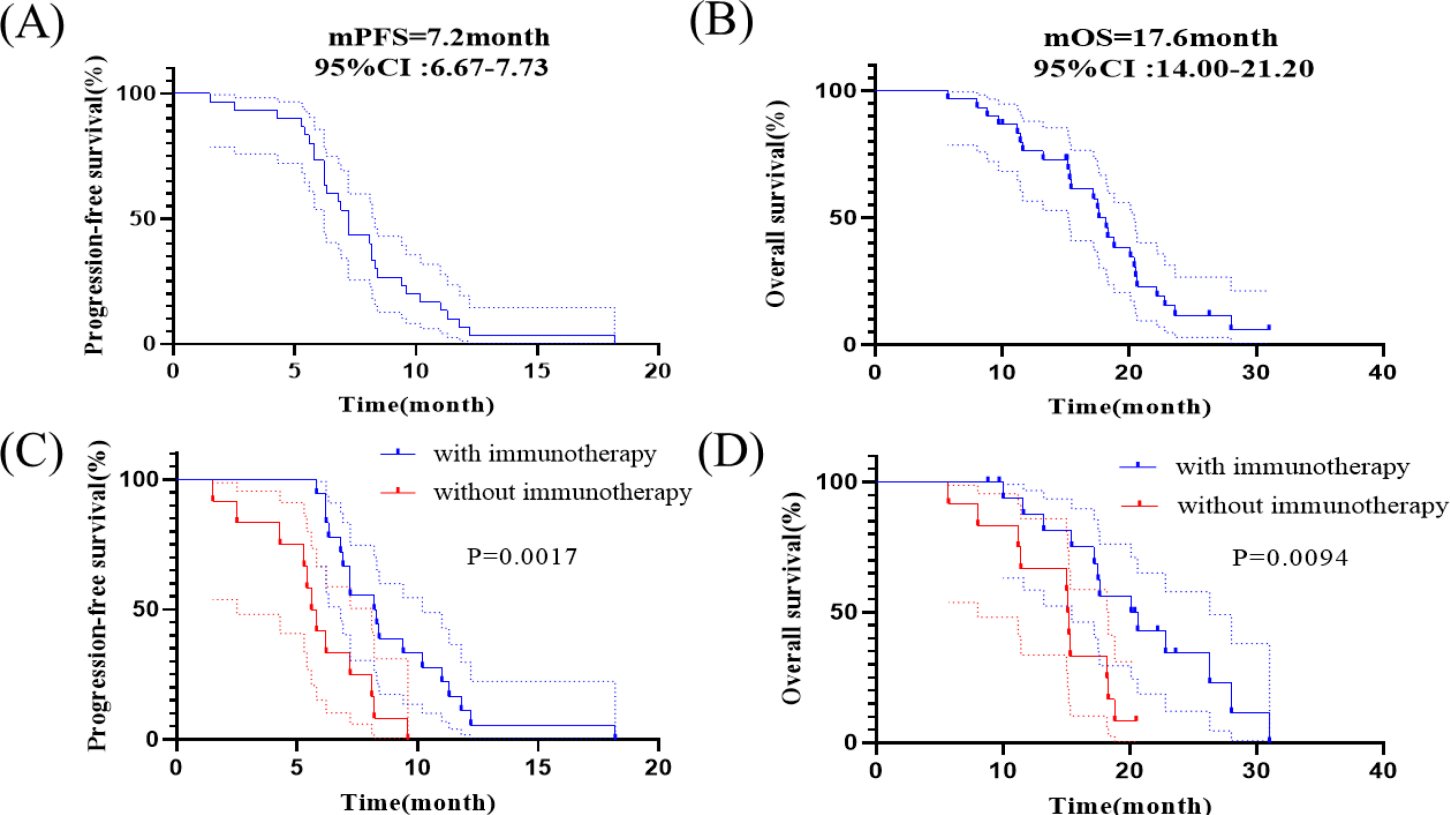
Kaplan-Meier analyses of survival. **(A)** Kaplan-Meier curves of PFS in all patients. **(B)** Kaplan-Meier curves of OS in all patients. **(C)** Kaplan–­Meier curves of PFS in patients receiving anlotinib with or without immunotherapy. **(D)** Kaplan–­Meier curves of OS in patients receiving anlotinib with or without immunotherapy.

**Figure 3** shows the Kaplan - Meier curves of PFS and OS for 30 patients. The median PFS was 7.2 months (95% CI: 6.67-7.73 months) for all patients. The median OS for all patients was 17.6 months (95% CI: 14.00-21.20 months), the 1-year OS rate is 76.7% (**Figures 3A, B**). The median PFS was 8.2 months (95% CI: 5.91-10.49 months) for patients who received synchronized immunotherapy and 5.6 months (95% CI: 4.92-6.28 months) for patients who did not receive synchronized immunotherapy, which presented statistically significant difference between the two groups, *p*=0.0017 (**Figure 3C**). The median OS was 20.1 months (95% CI: 14.61-25.59 months) for patients who received synchronized immunotherapy and 15.1 months (95% CI: 14.76-15.44 months) for patients who did not receive synchronized immunotherapy, which presented statistically significant difference between the two groups, *p*=0.0094 (**Figure 3D**). We analyzed the risk factors associated with OS through COX regression model (**Table 4**). Univariate analysis showed that neoplasm staging (*p*=0.042) and the application of combined immunotherapy (*p*=0.014) were significantly associated with OS. In multivariate analysis, neoplasm staging (IV vs. III, HR=2.71 95% CI:1.04-7.07 *p*=0.041) and the application of combined immunotherapy (with immunotherapy vs. with chemotherapy, HR=0.30 95% CI:0.12-0.78 *p*=0.014) were two independent risk factors for OS.

**Table 4:** Cox multivariate analysis of Overall survival (OS) in all patients (n=30).

	Univariate analysis		Multivariate analysis	
Variables	*p*	HR [95%CI]	*p*	HR [95%CI]
Sex (male vs female)	0.304	0.45 [0.10,2.06]	0.173	0.34 [0.07,1.61]
Age group (≥65 vs. <65)	0.357	0.68 [0.30,1.55]		
ECOG PS at baseline				
0				
1	0.665	1.31 [0.39,4.40]		
2	0.993	1.00 [0.31,3.26]		
Smoking history (Yes vs. No)	0.453	1.43 [0.56,3.66]		
Hypertension history (Yes vs. No)	0.993	1.00 [0.41,2.44]		
Neoplasm staging (IV vs. III)	0.042	2.67 [1.04,6.87]	0.041	2.71 [1.04,7.07]
Lymph node metastasis (Yes vs. No)	0.794	0.87 [0.29,2.58]		
Anlotinib in therapy line (2nd line vs. 1st line)	0.761	1.26 [0.29,5.46]		
Anlotinib with immunotherapy (Yes vs. No)	0.014	0.31 [0.12,0.79]	0.014	0.30 [0.12,0.78]

**Figure 4:**
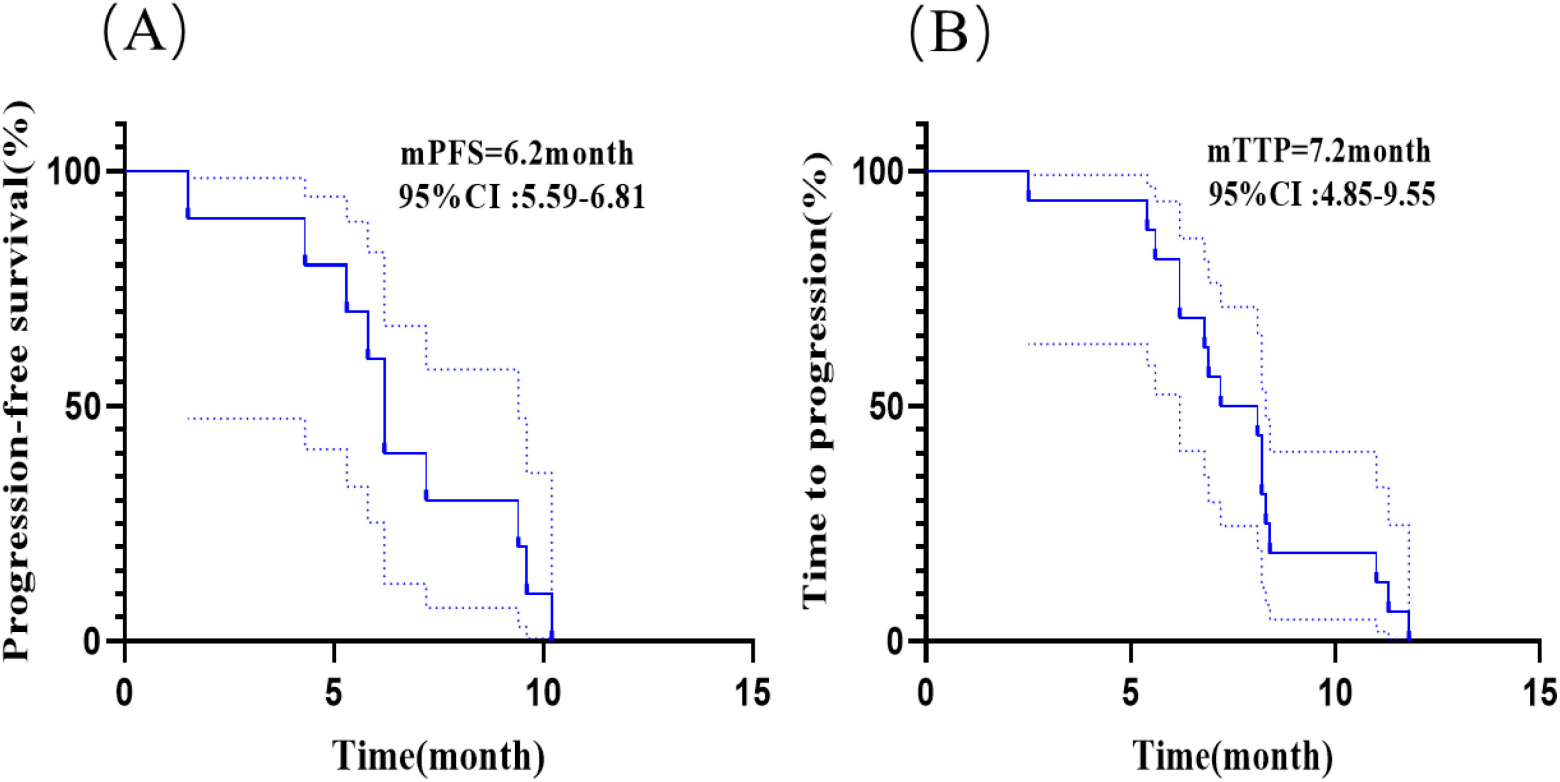
Kaplan-Meier analyses in patients with brain metastases. **(A)** Kaplan-Meier curves of PFS for patients who had brain metastases before receiving anlotinib. **(B)** Kaplan-Meier curves of time to intracranial progression (TTP) for patients who developed brain metastases after receiving anlotinib.

In this study, 33.3% (10/30) of all patients had brain metastases before receiving anlotinib; 53.3% (16/30) of all patients developed brain metastases after receiving anlotinib including 10 patients who received anlotinib in combination with immunotherapy; 13.3% (4/30) of all patients did not develop brain metastases during the course of the disease. The **Figure 4A** showed the median PFS was 6.2 month (95% CI: 5.59-6.81 months) for patients who had brain metastases before receiving anlotinib. The **Figure 4B** showed the median time to intracranial progression (TTP) was 7.2 months (95% CI: 4.85-9.55 months) for patients who developed brain metastases after receiving anlotinib. The median time to intracranial progression was not statistically different between the two groups of patients with or without immunotherapy.

### Treatment-related adverse events

According to CTCAE5.0, all 30 enrolled patients were included in the safety analysis, and only anlotinib associated adverse events were analyzed. **Table 5** shows most AEs were minor (grade 1-2) and manageable. The most common grade 1-2 AEs were anemia (14/30, 46.7%), hypertension (13/30, 43.3%), elevated transaminases (11/37, 36.7%) and leukopenia (11/30, 36.7%). Grade 3-4 AEs were reported in 10 patients (33.3%) including 2 patients with severe anemia, 1 patient with grade 3 hypertension, and transaminase elevation in 3 cases, leukopenia in 2 cases, neutropenia in 1 case and severe fatigue in 1 case. Reduction or temporary discontinuation of anlotinib were administered to all the patients experienced adverse events of grade 3-4. No unexpected AEs were observed, and no treatment-related deaths occurred in this study.

**Table 5:** Treatment- related adverse events with anlotinib.

Adverse events	All grades [no. (%)]	≥Grade 3 [no. (%)]
Anemia	14 (46.7)	2 (6.7)
Hypertension	13 (43.3)	1 (3.3)
Elevated transaminases	11 (36.7)	3 (10.0)
Leukopenia	11 (36.7)	2 (6.7)
Neutropenia	9 (30.0)	1 (3.3)
Elevated white blood cells	8 (26.7)	0 (0)
Fatigue	8 (26.7)	1 (3.3)
Elevated alkaline phosphatase	5 (16.7)	0 (0)
Thrombocytopenia	5 (16.7)	0 (0)
Weight loss	5 (16.7)	0 (0)
Thrombosis	4 (13.3)	0 (0)
Proteinuria	4 (13.3)	0 (0)
Decreased appetite	4 (13.3)	0 (0)
Hand-foot syndrome	3 (10.0)	0 (0)
Hemorrhage	2 (6.7)	0 (0)
Constipation	2 (6.7)	0 (0)
Vomiting	2 (6.7)	0 (0)
Hypothyroidism	2 (6.7)	0 (0)
Rash	1 (3.3)	0 (0)
Nausea	1 (3.3)	0 (0)
Diarrhea	1 (3.3)	0 (0)
Hypocalcemia	1 (3.3)	0 (0)
Hyperkalemia	1 (3.3)	0 (0)

## Discussion

Currently, the main treatment methods for ES-SCLC include systemic chemotherapy, local radiotherapy, targeted therapy and immunotherapy. However, with conventional first-line etoposide/platinum chemotherapy, most ES-SCLC patients relapse rapidly with a median PFS of less than 6 months and a median OS limited to approximately 10 months (14). Meanwhile, because of the lack of effective maintenance therapy methods for patients who have received standard chemotherapy, many patients will suffer from rapid advancement of disease which leads to poor prognosis. Therefore, the treatment of ES-SCLC has been a major challenge for oncologists and new therapeutic drugs and patterns are under exploration.

By blocking tumor neovascularization, tumor cells tend to be degenerated and necrotic due to ischemia and hypoxia which could inhibit the development of tumor. In recent years, many drugs that inhibit tumor angiogenesis in the treatment of ES-SCLC have shown their efficacy but the results are not satisfactory. Endo and bevacizumab are widely used anti-angiogenic drugs in clinical practice. In the SALUTE II trial which evaluated the efficacy of standard chemotherapy plus bevacizumab in ES-SCLC, patients in the etoposide-platinum-bevacizumab combination group had a longer median PFS (5.5m vs. 4.4m) comparing with the patients in the etoposide-platinum alone group. However, no statistical difference in median OS was observed (9.4m vs. 10.9m) between the two groups (15). In a multicenter, randomized controlled study, it was found that the addition of rh-endostatin (recombinant human endothelial inhibitor) to first-line chemotherapy did not significantly improve PFS (6.4 vs. 5.9 months, *p*=0.2126), OS (12.1 vs. 12.4 months, *p*=0.8119) and ORR (75.4% vs. 66.7%, *p*=0.3483) (16).

Anlotinib, a multi-targeted TKI, is a novel anti-tumor drug developed by China which can effectively inhibit the effects of VEGF/FGFR/PDGF-BB mediated regulatory pathway of tumor angiogenesis. The ALTER1202 study evaluated the efficacy of anlotinib in the third-line treatment of SCLC patients. In the 120 patients enrolled, the anlotinib group (n=81) gained significantly prolonged PFS by 3.4 months (4.1 m vs. 0.7 months, *p*<0.0001) and OS by 2.4 months (7.3 m vs. 4.9 m, *p*=0.0029) comparing with the placebo group (n=38). The DCR in the anlotinib group was also significantly higher than that in the placebo group (71.6% vs. 13.2%) (17). Based on this study, anlotinib has been approved for the third-line treatment of progressive or relapsed SCLC patients in China.

Our single-armed retrospective study revealed that all patients receiving anlotinib as maintenance therapy in the first-line or second-line therapy gained the median PFS of 7.2 months and median OS of 17.6 months, the ORR and DCR were 50.0% and 86.7%. Meanwhile, the median PFS and median OS were 5.6 months and 15.1 months respectively for patients receiving anlotinib in combination with chemotherapy. In a clinical trial conducted by Deng P et al., the median PFS and OS were 8.02 months and 15.87 months in the 35 patients with ES-SCLC receiving anlotinib plus platinum-etoposide as first-line treatment (18). Another similar retrospective study led by Zheng H et al. revealed that the median PFS and OS are 6.0 months and 10.5 months respectively in 58 patients included in this study (19). Therefore, the results in our study are similar and comparable to the recent similar researches which presents a promising application of anlotinib in combination of chemotherapy.

SCLC has long been considered immunogenic with a very high genetic mutation rate. Previous studies have shown that the efficacy of immunotherapy is positively correlated with tumor mutation burden (TMB) which indicates that immunotherapy is a viable treatment for small cell lung cancer (20). A multicenter phase II study evaluated the efficacy of Ipilimumab in combination with carboplatin and etoposide as first-line chemotherapy for patients with ES-SCLC, indicating that the immune-related ORR reached to 84.8%, the median PFS and OS were 6.9 months and 17.0 months respectively (21). Meanwhile, another two studies including Impower133 and CASPIAN showed that the addition of PD-L1 inhibitors like atezolizumab and durvalumab with platinum-etoposide chemotherapy could both improve the median OS to 12.3 months and 13.0 months respectively comparing with the chemotherapy alone group.

The clinical evidence about anti-angiogenic therapy combined with immunotherapy in SCLC is limited. In a phase Ib trial in 22 patients with advanced NSCLC, sintilimab plus anlotinib as first-line therapy achieved an ORR of 72.7% and a DCR of 100%, with a PFS of 15 months. The rate of grade 3 or higher AEs (most commonly hypertension) was 54.5% (22). A multicenter study of camrelizumab in combination with apatinib in the second-line treatment of ES-SCLC showed good efficacy and acceptable AEs in patients with relapsed SCLC (23). Our study also found that the median PFS and OS were 8.2 months and 20.1 months respectively for the patients with ES-SCLC receiving anlotinib plus immunotherapy including PD-1 or PD-L1 inhibitors, which presented statistically significant differences comparing with chemotherapy group. This result showed better efficacy compared with traditional chemotherapy or anlotinib plus chemotherapy, which proved that anlotinib combined with immunotherapy might better benefit patients for the survival. The multivariate analysis also demonstrated that anlotinib plus immunotherapy could independently influence the OS of the patients with ES-SCLC. Since there was no control group in our study, the efficacy of this combination therapy still requires further verification by prospective studies with larger sample.

Brain metastases always indicates poor prognosis for patients with ES-SCLC during the course of treatment. The effective therapeutic patterns to delay the brain metastases or intracranial progression are under exploration. ALTER1202 trial which evaluated anlotinib in patients with ES-SCLC combined with stable brain metastases in the third-line and post-line therapy found that anlotinib alone (n=21) versus placebo (n=9) had a PFS of 3.8m versus 0.8m (HR = 0.15) (17). It indicated that anlotinib had a control effect on stable brain metastases in SCLC. In a recent retrospective study researching on the progression free survival benefit of SCLC patients treated with anlotinib as second or later line treatment found that patients with or without brain metastases before receiving anlotinib therapy gained the median PFS of 2.5 months and 1.5 months respectively (24). Another single-arm trial investigating anlotinib plus platinum-etoposide as a first-line treatment for ES-SCLC revealed that median PFS in patients with brain metastases was 7.34 months (18). Our study showed that the median PFS was 6.2 months for patients with brain metastases before receiving anlotinib based therapy and the median time to intracranial progression reached to 7.2 months for patients without brain metastases before the anlotinib based therapy. Therefore, comparing with previous studies, anlotinib as maintenance therapy can significantly prolong the time of intracranial progression for patients without initial brain metastases and it can also improve the median PFS and achieve good intracranial control for patients with initial brain metastases.

In our study, the anlotinib based combination regimen related AEs were all acceptably tolerated. No unexpected toxicity was observed and no treatment-related deaths occurred. The most common AEs were anemia, hypertension, elevated transaminases and leukopenia. Grade 3-4 adverse events included severe anemia, hypertension, transaminase elevation, leukopenia, neutropenia and severe fatigue. Similar to previous studies, bone marrow suppression was the most common adverse events (12, 13, 18). No new anlotinib-related AEs were observed in this study and the toxicity profile was similar to other studies of anlotinib in SCLC (17). Reduction or temporary discontinuation of anlotinib were administered to all the patients experienced adverse events of grade 3-4. Due to the bias of retrospective studies, the incidence of AEs in this study may be lower than the actual data in the real world and some AEs may be influenced by concurrent immunotherapy or chemotherapy.

Our study has indicated the good efficacy and safety about the application of anlotinib in the combination therapy and subsequent maintenance therapy in the first-line or second-line treatment of ES-SCLC. However, there are still some limitations of our study. The relatively small sample size of our retrospective single-center study resulted in no enough available data for several subgroups, which may reduce the statistical power. Large prospective studies are needed to confirm our results. Meanwhile, comparisons within chemotherapy alone or immunotherapy alone were missing in this single-arm study and a multicenter randomized controlled trial is desired to provide additional evidence.

In summary, anlotinib is an effective option as second-line or prior-line maintenance therapy with good efficacy and tolerable adverse reactions for ES-SCLC patients. We revealed that patients were sensitive to the therapeutic regimen and gained prolonged PFS and OS, especially when patients received the therapy of anlotinib plus immunotherapy. Future larger randomized clinical studies are needed to further confirm the efficacy of anlotinib in the treatment of ES-SCLC.

## Data Availability

The original contributions presented in the study are included in the article/supplementary material. Further inquiries can be directed to the corresponding author.
